# Perceived Stress Among Private School Teachers in Chengalpattu District, Tamil Nadu, India: A Cross-Sectional Study

**DOI:** 10.7759/cureus.71848

**Published:** 2024-10-19

**Authors:** Lavanya M, Pradeep MVM, Anantharaman VV, Logaraj M

**Affiliations:** 1 Community Medicine, SRM Medical College Hospital and Research Centre, Kattankulathur, IND

**Keywords:** india, perceived stress, pss-10, tamilnadu, teachers

## Abstract

Background

Teachers are the cornerstone of any civilized society, preserving knowledge, wisdom, and ideals. Along with job-related stress, teachers face a variety of challenging circumstances in the classroom. This includes thoughts concerning a lack of professional appreciation, challenges maintaining classroom discipline, a lack of peer support, and extra duties like surveys and government mandates. All of these characteristics may have an effect on their mental health. Thus the objective of the study is to analyze the perceived stress among private school teachers in Chengalpattu district and associate the findings with various demographic and occupational parameters.

Methodology

A cross-sectional study was conducted among 300 private school teachers in Chengalpattu District, Tamil Nadu from June to August 2024. The principal investigator collected the data by using a semi-structured questionnaire, which consists of three parts, that is, sociodemographic variables, questions about their profession and health, and stress assessed by using the Perceived Stress Scale (PSS-10). The data collected was entered in Microsoft Excel (Microsoft Corporation, Redmond, Washington, USA) and analyzed using IBM SPSS Statistics for Windows, Version 27.0 (Released 2019; IBM Corp., Armonk, New York, USA)

Results

The current study included 300 teachers from private schools. Majority of teachers (n=152, 50.7%) were over the age of forty. Of the participants, 232 (77.3%) were female, and 122 (40.7%) reported having trouble sleeping. Comorbidities were present in nearly 79.7% (239/300) of teachers. In this study, during the last month preceding the survey, 85% (255/300) of the teachers reported feeling anxious and stressed ("fairly" or "very often") and 72% (217/300) felt they were not able to control significant things in their lives. According to the PSS-10, the majority of individuals (n=223, 74.3%) experienced high levels of stress, followed by low stress (n=45, 15%) and moderate stress (n=32, 10.7%). The mean PSS score in this study was 26.50±9.92. Inferential statistics revealed that teachers who were aged more than 40, those with a monthly salary of less than or equal to Rs.40000, smokers, married, teachers with an average of more than 40 students per class, teaching experience of more than 20 years, those who had disturbed sleep, and those with comorbidities experienced high stress according to the PSS-10. Gender, alcohol consumption habits, the duration of schoolwork accomplished at home per day, and workplace/home conflict all had no significant association with perceived stress.

Conclusion

In addition to providing programs that promote or educate teachers about occupational risks, administrators in education and legislators should be mindful of the physical and psychological conditions of teachers and should also offer a wide range of preventive strategies that protect teachers' mental health and improve their social functioning.

## Introduction

Teachers are the cornerstone of any civilized society, preserving knowledge, wisdom, and ideals. In a large country like India, with such a diverse and ignorant population, teachers are frequently a child's sole source of learning. Teaching is considered one of the most important vocations in the world. Teachers have always played a vital role in the development of civilization. Aside from teaching, teachers spend many hours per week in classroom activities. Teachers often participate in other extracurricular endeavors (for example, preparing and correcting tests and serving as school administrators) resulting in an immense workload [1]. Furthermore, teachers struggle to reduce working hours due to efforts to improve educational excellence and societal norms [2]. Along with job-related stress, teachers face a variety of challenging circumstances in the classroom [3]. This includes thoughts concerning a lack of professional appreciation, challenges maintaining classroom discipline, a lack of peer support, and extra duties like surveys and government mandates. All of these characteristics may have an effect on their mental health.

Another qualitative study found that teachers are stressed due to a shortage of resources, constant oversight, professional disinterest and fatigue, competitive edge and goals, students' detrimental or reckless conduct, high expectations and requirements, and a lack of leisure time among various other variables [4]. Because of their multitasking, school teachers are more likely to engage in "emotional labor". The emotional crossover between teachers and students was significantly influenced by teachers' stress from poor-quality sleep, which, in turn, adversely affected students' academic motivation and satisfaction in the classroom. Social, psychological, and physical motives all affect the workplace and can lead to burnout, which is harmful to one's physical and mental well-being. In addition to being a major occupational health concern that can cost employers a lot of money, mental health problems can cause workers to be absent from work [5]. Noncommunicable diseases (NCDs) such as diabetes, cancer, heart diseases, cerebrovascular accident, and chronic lung diseases account for 74% of all fatalities globally [6]. Schoolteachers are prone to hypertension due to their stressful occupations and sedentary lifestyles [7].

Teachers in private schools experience distinct obligations than those in government schools. Some of these differences include managing an extensive student body, teaching more classes per day, performing tasks for more hours, and not obtaining enough downtime from the classroom. Other aspects to take into account are age, gender, and the number of years of teaching expertise. Therefore, it is essential to comprehend the mental health of private school teachers because doing so will improve their working conditions and general well-being. It may be possible to improve or modify the satisfactory working atmosphere for private school teachers by considering both the positive and negative aspects of their perceived stress. Thus the objective of the study is to analyze the perceived stress among private school teachers and associate the findings with various demographic and occupational parameters.

## Materials and methods

Study settings and duration

A cross-sectional study was conducted among private school teachers in Chengalpattu District, Tamil Nadu from June to August 2024.

Sample size calculation and sampling method

Based on a comprehensive review of the literature, the prevalence of moderate stress according to PSS-10 among school teachers was 77.20% [8]. The sample size was derived using the formula N=3.84 * p * q / d2, where p represents the prevalence rate, q represents the complement of p, and d represents the accuracy with a 5% absolute error. The formula yielded a sample number of 282. The sample size was rounded off to 300. Nonrespondents were not included in the study. A multistage random sampling technique was used to determine the sample size. Chengalpattu district consists of eight blocks. Simple random sampling was used to select Kattankulathur and St Thomas Mount blocks from eight in Chengalpattu district. The list of the number of matriculation schools in both blocks was obtained from the district's Chief Education Officer. Kattankulathur block and St Thomas Mount block comprise 35 and 103 matriculation schools, respectively. Two schools were chosen from each block by lottery method. The number of teachers recruited for the study from each school was decided using the probability proportionate to size. The Principal or Chairperson of the chosen school was notified of the visit via phone and provided information about the study's purpose and significance.

Inclusion criteria

Participants included both male and female private school teachers who were teaching students in grades 8 through 12 and those who were more than twenty years old and provided their consent. After being informed about the study, the teachers were required to sign a consent form indicating that they would willingly take part.

Exclusion criteria

Primary and middle school educators were not permitted to participate in the study since the relevant authorities had not granted their approval, hence they were excluded.

Data collection procedure

The lead investigator performed in-person interviews in order to gather data. To maintain secrecy, each interview was held in a private environment and lasted roughly 20 minutes. A self-reporting semi-structured questionnaire was used consisting of three sections.

Study tools

A) Their sociodemographic profile was the focal point of the first section of the questionnaire. B) The second section had questions about their profession and health. C) Measure of perceived stress: The Perceived Stress Scale-10 (PSS-10) was used extensively in the third section and has been psychometrically verified as a reliable tool for assessing psychological stress that has occurred in the last four weeks [9]. Ten areas make up the survey, and each is graded on a five-point Likert scale (0 being "never" and 4 being "very often")."General stressors" is the first factor, and "the ability to cope" is the second in the dual-component structure of the PSS-10 construct [10]. Items 4, 5, 7, and 8 include positive expressions, hence their scores are reverse-coded. The total of all the item scores determines the PSS-10 score. Higher felt stress was reflected in higher scores. There were ten questions in all, with a 40-point maximum and a 0-point minimum. 0-13 indicates low stress, 14-26 denotes moderate stress and 27-40 reflects high perceived stress [11]. There is no diagnostic threshold on the PSS-10 that distinguishes between people who are stressed and those who are not [12].

Statistical analysis

The data was entered in Microsoft Excel (Microsoft Corporation, Redmond, Washington, USA) and the results were analyzed using IBM SPSS Statistics for Windows, Version 27.0 (Released 2019; IBM Corp., Armonk, New York, USA). Frequency and proportion were used to represent categorical variables. By utilizing inferential statistics like the chi-square test to analyze the data, a statistically significant difference between discrete variables in the two groups was determined. P value <0.05 was considered to be statistically significant.

## Results

The current study included 300 teachers from private schools. The majority of teachers (50.7%) were over the age of forty. There were 68 male teaching staff (22.7 %) and 232 female teachers (77.3%). Out of the participants, 237 teachers were married. About 174 teachers (58%) have a monthly income of less than ≤Rs.40000. Most of the teachers (54.3%) had ≤20 years of experience. Of the 300 schoolteachers, 253 (84.3%) reported doing schoolwork at home for more than 45 minutes each day. Of them, 122 (40.7%) reported having trouble sleeping. The majority of teachers reported no conflict at work or at home. Comorbidities were present in nearly 79.7% of teachers. Table 1 illustrates the background details, teaching, and health-related characteristics of study participants.

**Table 1:** Sociodemographic distribution of study participants (n=300)

Variables		Frequency	Percentage
Age	≤40 years	148	49.3
	>40 years	152	50.7
Gender	Male	68	22.7
	Female	232	77.3
Income per month	≤Rs.40000	174	58
	>Rs.40000	126	42
Marital status	Married	237	21
	Unmarried	63	21.3
Smoking	Yes	38	12.7
	No	262	87.3
Alcohol intake	Yes	26	8.7
	No	274	91.3
Teaching experience in years	≤20 years	163	54.3
	>20 years	137	45.7
Average students per class	≤40	101	33.7
	>40	199	66.3
Duration of school work done at home per day	≤45 mins	47	15.7
	>45 mins	253	84.3
Disturbed sleep	Present	122	40.7
	Absent	178	59.3
Conflict at workplace/home	Present	75	25
	Absent	225	75
Comorbidities	Present	239	79.7
	Absent	61	20.3

The participant's degree of stress was evaluated based on the events they encountered in the previous month using the PSS-10 questionnaire. During the last month preceding the survey, 85% (255/300) of the teachers reported feeling anxious and stressed ("fairly" or "very often") and 72% (217/300) felt they were not able to control significant things in their lives (Table 2).

**Table 2:** Responses of the participants to questions of PSS-10 (n=300) PSS: Perceived Stress Scale

Items	Never, n (%)	Almost never, n (%)	Sometimes, n (%)	Fairly often, n (%)	Very often, n (%)
1. In the last month, how often have you been upset because of something that happened unexpectedly?	43 (14.3)	45 (15)	65 (21.7)	77 (25.7)	70 (23.3)
2. In the last month, how often have you felt that you were unable to control the important things in your life?	16 (5.3)	9 (3)	58 (19.3)	143 (47.7)	74 (24.7)
3. In the last month, how often have you felt nervous and “stressed”?	8 (2.7)	28 (9.3)	9 (3)	108 (36)	147 (49)
4. In the last month, how often have you felt confident about your ability to handle your personal problems?	24 (8)	160 (53.3)	46 (15.3)	43 (14.3)	27 (9)
5. In the last month, how often have you felt that things were going your way?	24 (8)	193 (64.3)	12 (4)	43 (14.3)	28 (9.3)
6. In the last month, how often have you found that you could not cope with all the things that you had to do?	8 (2.7)	63 (21)	73 (24.3)	70 (23.3)	86 (28.7)
7. In the last month, how often have you been able to control irritations in your life?	43 (14.3)	186 (62)	26 (8.7)	37 (12.3)	8 (2.7)
8. In the last month, how often have you felt that you were on top of things?	24 (8)	205 (68.3)	26 (8.7)	35 (11.7)	10 (3.3)
9. In the last month, how often have you been angered because of things that were outside of your control?	8 (2.7)	54 (18)	82 (27.3)	16 (5.3)	140 (46.7)
10. In the last month, how often have you felt difficulties were piling up so high that you could not overcome them?	28 (9.3)	43 (14.3)	48 (16)	64 (21.3)	117 (39)

**Figure 1:**
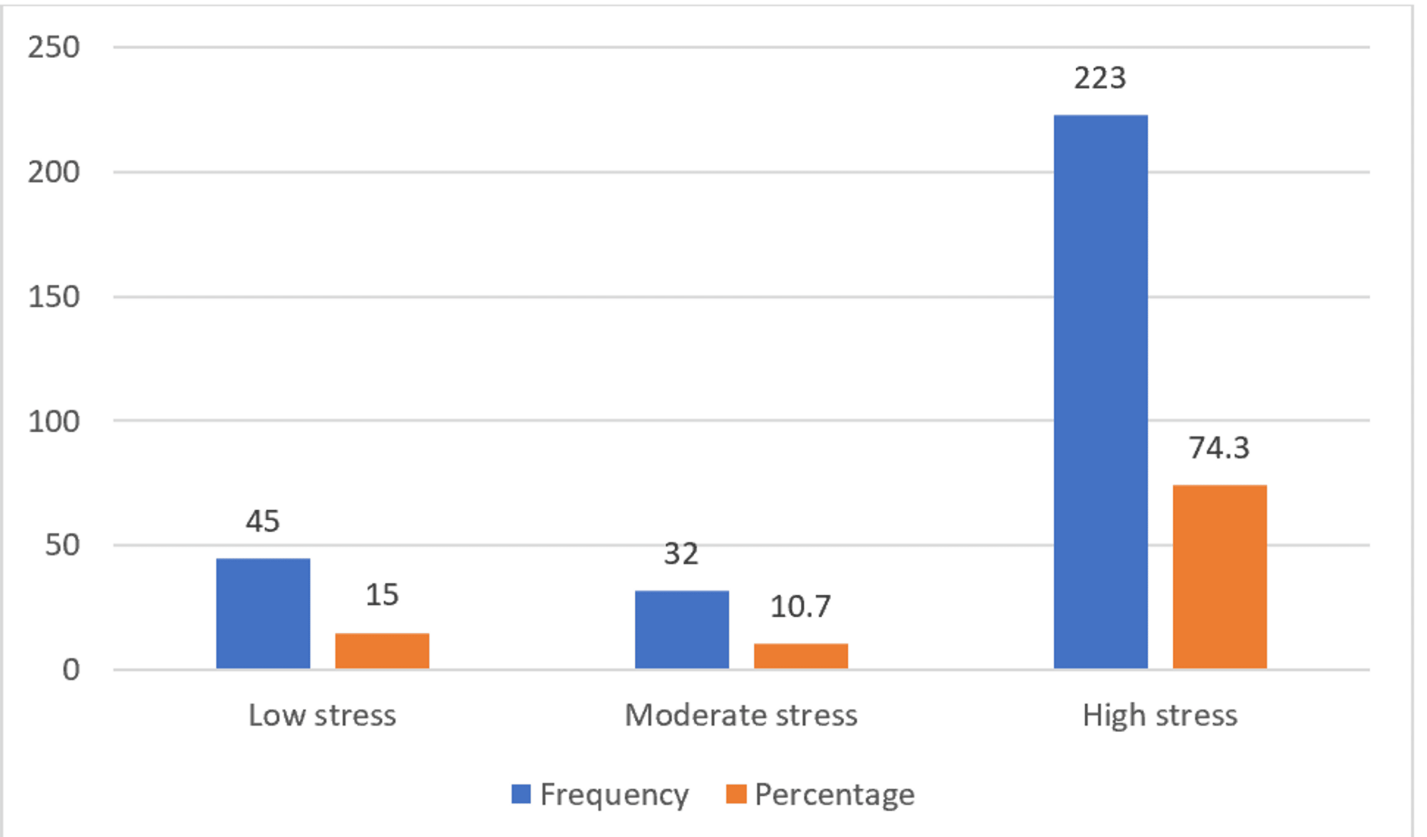
Bar diagram displaying study participants’ distribution according to PSS-10 categories (n=300) Scores 0-13 indicate low stress, 14-26 denote moderate stress, and 27-40 reflect high perceived stress. PSS: Perceived Stress Scale

The study participants’ distribution according to PSS-10 categories is shown in Figure 1. According to the PSS-10, the majority of individuals (n=223, 74.3%) experienced high levels of stress, followed by low stress (n=45, 15%) and moderate stress (n=32, 10.7%). The mean PSS score in this study was 26.50±9.92.

Table 3 displays the association between several sociodemographic characteristics and PSS-10 categories. A significant association (p≤0.001) was determined between age and perceived stress levels, with those over 40 (78.3%) experiencing greater stress than those under 40. Those with a monthly salary of less than or equal to Rs.40000 showed a higher percentage of stress (79.9%), which was statistically significant by the chi-square test (p≤0.001). Married individuals (81%) experienced more stress than unmarried people. There was a significant difference in PSS-10 proportions based on marital status (p<0.001). Habits of smoking (p=0.02) were significantly associated with reported stress levels, with smokers perceiving higher stress (92.1%). Teachers with over 20 years of experience had significantly higher stress levels (76.6%, p<0.001). There was a significant association between stress levels and average pupils per class (p≤0.001). Teachers with an average of more than 40 students per class reported higher stress levels (76.9%). Disrupted sleep (p=0.022) was significantly associated with feelings of stress levels, with 79.5% reporting heightened stress levels. Teachers with comorbidities experienced significantly greater levels of stress (82%), as indicated by the chi-square test (p≤0.001). Gender, alcohol consumption habits, the duration of schoolwork accomplished at home per day, and workplace/home conflict all had no significant association with perceived stress.

**Table 3:** Association between various sociodemographic variables and PSS categories ^*^p-value <0.05 is considered to be statistically significant. Scores 0-13 indicate low stress, 14-26 denote moderate stress, and 27-40 reflect high perceived stress. PSS: Perceived Stress Scale

Variables		Low stress, n (%)	Moderate stress, n (%)	High stress, n (%)	Total (n=300)	Chi-square (p-value)
Age	≤40 years	40 (27)	4 (2.7)	104 (70.3)	148	46.18 (<0.001)*
	>40 years	5 (3.3)	28 (18.4)	119 (78.3)	152	
Gender	Male	8 (11.8)	8 (11.8)	52 (76.5)	68	0.76 (0.68)
	Female	37 (15.9)	24 (10.3)	171 (73.7)	232	
Income per month	≤Rs.40000	30 (17.2)	5 (2.9)	139 (79.9)	174	26.69 (<0.001)*
	>Rs.40000	15 (11.9)	27 (21.4)	84 (66.7)	126	
Marital status	Married	16 (6.8)	29 (12.2)	192 (81)	237	60.57 (<0.001)*
	Unmarried	29 (46)	3(4.8)	31 (49.2)	63	
Smoking	Yes	1 (2.6)	2 (5.3)	35 (92.1)	38	7.47 (0.02)*
	No	44 (16.8)	30 (11.5)	188 (71.8)	262	
Alcohol intake	Yes	4 (15.4)	3 (11.5)	19 (73.1)	26	0.029 (1.0)
	No	41 (15)	29 (10.6)	204 (74.5)	274	
Teaching experience in years	≤20 years	40 (24.5)	5 (3.1)	118 (72.4)	163	41.16 (<0.001)*
	>20 years	5 (3.6)	27 (19.7)	105 (76.6)	137	
Average students per class	≤40	26 (25.7)	5 (5)	70 (69.3)	101	16.89 (<0.001)*
	>40	19 (9.5)	27 (13.6)	153 (76.9)	199	
Duration of school work done at home per day	≤45 mins	8 (17)	7 (14.9)	32 (68.1)	47	1.37 (0.50)
	>45 mins	37 (14.6)	25 (9.9)	191(75.5)	253	
Disturbed sleep	Present	10 (8.2)	15 (12.3)	97 (79.5)	122	7.59 (0.022)*
	Absent	35 (19.7)	17 (9.6)	126 (70.8)	178	
Conflict at workplace/home	Present	14 (18.7)	4 (5.3)	57 (76)	75	3.6 (0.16)
	Absent	31 (13.8)	28 (12.4)	166 (73.8)	225	
Comorbidities	Present	19 (7.9)	24 (10)	196 (82)	61	48.69 (<0.001)*
	Absent	26 (42.6)	8 (13.1)	27 (44.3)	239	

## Discussion

This is a cross-sectional study conducted among 300 private school teachers in Chengalpattu district. The study participants were put into categories based on their PSS-10 ratings, with a mean score of 26.50±9.92. These data indicate that, on average, study participants reported high levels of perceived stress. However, there was a substantial variation in stress levels among people in the study population. Our study revealed that the majority of teachers (74.3%) reported high levels of perceived stress, which is identical to a study done among secondary school teachers in Uganda, which found that the majority of teachers (60.2%) experienced heightened levels of stress [13]. The frequency of various stress levels among study participants is highlighted by this data, underscoring the significant impact that perceived stress has on people's mental and physical health. According to a previous study, male teachers experience significantly higher levels of stress at work than female teachers [14]. This finding concurs with our research and differs from those of other studies [15-17]. Male teachers may be more stressed out than female teachers because of the stigma associated with mental illness. Men are frequently less inclined to seek treatment for mental health problems because of this stigma, which raises stress levels.

A study done among among urban Indian school teachers showed that occupational stress is largely influenced by demographic and work-related characteristics including age and experience [18]. This result matches up with the findings of our study. This can be because senior teachers frequently take on leadership positions, like department chiefs or guides, which adds to their workload and stress. Teachers who have been teaching for several years may be expected to perform better and be held to higher standards. Teachers may suffer from chronic stress and tiredness after decades of teaching, which can result in burnout and fatigue. According to the results of our study, 79.9% of people with lower salaries reported feeling more stressed. Reduced pay may cause financial difficulty, which in turn may exacerbate stress. Similar to another study, this study found that married teachers faced more occupational stress compared to unmarried teachers [19]. Family-related stresses including juggling job and family, having more financial obligations, and having relationship difficulties could be the cause of this. Similar to the current study, the former study revealed that an increase in the number of students in a class significantly drove teachers' greater stress levels [20]. Larger class sizes may be the cause of this, as they result in an extremely intense workload and difficulties with classroom management, which in turn raise teacher stress levels. Compared to other professions, teaching entails greater levels of stress, worry, exhaustion, and sleep issues [21].

According to the current study, teachers who encountered sleep disturbances also had greater levels of stress. Effective conflict management techniques can help lessen the negative effects of interpersonal conflict among coworkers, which can have a substantial impact on teachers' stress [22]. This finding contradicts the findings of our study since there was no discernible correlation between the perceived stress category and conflict at home or at work. Our study revealed that teachers with comorbidities experienced high levels of stress according to the PSS-10. This finding is consistent with another study conducted among 400 school teachers in Andhra Pradesh, which found that variables associated with an increased prevalence of prehypertension and hypertension encompassed perceived moderate/severe work stress [23]. Stress has been attributed to both the onset and worsening of diabetes [24]. In South African teachers, diabetes mellitus is linked to a higher incidence of psychological issues and depression [25]. Several medical conditions, including metabolic diseases, obesity, hypertension, and cardiovascular disorders have been associated with teaching.

Based on inferential statistics, it was found that teachers with comorbidities experienced high levels of stress according to the PSS-10, as did those who were older than 40, married, smokers, had more than 20 years of teaching experience, and had an average class size of more than 40 students. These teachers need to receive medical care. The findings suggest that a combination of behavioral, health-related, and sociodemographic factors influence how participants perceive stress. This highlights the level of complexity in determining stress levels in this specific population. Teachers can anticipate a diminution in work-related disorders if they embrace efficient health management measures that minimize work-related stress. To alleviate the stress, staff members ought to participate in educational workshops and training sessions. Employees need to comprehend both the nature of their work and its significance to society in order to mitigate stress. Their productivity shall improve if their degree of stress declines.

Strengths

The assessment of individuals' stress levels gains credibility when validated instruments like the PSS-10 are used. By concentrating on a high-risk occupational category, the study also tackles a significant public health concern regarding the perceived stress levels of teachers and provides information that could direct focused solutions to improve their mental health and well-being.

Limitations

This study contains a few drawbacks that should be taken into account, notwithstanding its positives. Selection bias has resulted from the selection of just two blocks and two schools per block, which may limit the generality of our findings to the whole research region. Another possible limitation is recall bias, where participants may have reported their feelings of stress inaccurately out of forgetfulness or wanting to appear more positive. The responses may have also been impacted by a bias towards social desire, especially in cases where the questions dealt with delicate topics like drinking and smoking. Furthermore, because of the cross-sectional design, it is more challenging to establish a causality relationship between the sociodemographic variables and the observed stress levels. Longitudinal study designs in the future may yield additional insights into these associations.

## Conclusions

In addition to providing programs that promote or educate teachers about occupational risks that they may encounter on a daily basis as part of their job, administrators in education and legislators should be mindful of the physical and psychological conditions of teachers. They should also offer a wide range of preventive strategies that protect teachers' mental health and improve their social functioning. Future studies must take into consideration variables such as pre-existing medical conditions, mental health, and surrounding factors like the workplace, as these may potentially impact the observed results. Frequent planned checkups, along with initiatives to reduce employment and behavioral risk factors, should be implemented for teachers. Particular focus needs to be placed on accomplishing appropriate ergonomics, which can lessen the likelihood of severe noncommunicable diseases and subsequent correlated rates of illness and mortality. Given that teachers shape the future of their country, it is imperative to examine their perceived stress levels and to include them in school health programs to protect their well-being.
